# Intraoperative Parameters of Comminuted Proximal Humerus Fractures: A Comparison Between Deltoid-Split and Deltopectoral Approaches

**DOI:** 10.7759/cureus.26443

**Published:** 2022-06-30

**Authors:** Janapamala V Kishore, Amit R Kale, Vishal Patil, Sachin Sonawane, Rupa Madhavi Kopparthi, Chiranjeevi Jani, Abhinay Vadlamudi

**Affiliations:** 1 Orthopaedics, Dr. D. Y. Patil Medical College, Hospital & Research Centre, Pimpri, Pune, IND; 2 Radiology, Dr. D. Y. Patil Medical College, Hospital & Research Centre, Pimpri, Pune, IND

**Keywords:** blood loss, intra-operative parameters, philos plate, delto-pectoral, deltoid split, proximal humerus

## Abstract

Background and objective

Many controversies exist in the literature regarding proximal humerus fractures treated with various surgical procedures. The chosen approach decides the level of comfort with which the surgeon can perform a particular surgery in orthopedics and the amount of reduction a surgeon can bring to a fractured bone. The purpose of this study was to compare variables such as time taken for surgery, achievement of reduction, and intraoperative blood loss between the two most frequently employed surgical approaches for proximal humerus in comminuted fractures: the deltoid-split (DS) approach and deltopectoral (DP) approach.

Methods

All patients with Neer III and IV types proximal humerus fractures treated with Proximal Humeral Interlocking System (PHILOS) plating from 2017 to 2020 were invited to participate in the study. The exclusion criteria were as follows: Neer type I and II fractures, pre-existing limb pathology, patient refusal or patient being unfit for surgery, and patient requiring a different modality of treatment like external fixator and pinning. After obtaining consent, the dark envelope method was used to randomize patients into one of the two treatment methods. The variables analyzed were time taken for the surgery, intraoperative blood loss, anatomical reduction in immediate postoperative X-ray, and complications. The results were analyzed and findings were recorded.

Results

A total of 42 patients were randomized into the two groups (22 DS, 20 DP; mean age of 44.85 years for DS and 49.61 years for DP). In terms of age, gender, and Neer fracture classification, the groups were comparable. The average blood loss estimated was less in the DS group compared to the DP group; however, the difference was not statistically significant. Intraoperative time was not significantly different between the DS and the DP groups. The surgeons were able to achieve a significantly higher anatomical reduction in the immediate postoperative X-ray with the DP approach compared to the DS approach. The complications (two in DS and two in DP) in either approach were equal in number although all of them were unique.

Conclusions

The proximal humerus fracture treatment with a PHILOS plate is considered to be a reliable option using either of the described approaches. Based on our findings, the choice of the approach has no impact on surgical time and blood loss. However, patients who were operated on with the DP approach fared better in terms of achieving reduction as assessed by immediate postoperative X-ray owing to limited exposure distally limited by the axillary nerve.

## Introduction

Proximal humerus fractures are one of the most common fractures and represent approximately 4% of all fractures and 26% of humerus fractures with a reported incidence rate of approximately 250/100,000 persons per year [1,2]. Most proximal humerus fractures in young adults result from high-energy trauma sustained during road traffic accidents (RTAs), sports injuries, gunshot wounds, and falls from height, while elderly persons usually have a history of low-energy trauma sustained via simple falls with direct impact on the shoulder [3,4], or indirect impact because of a fall onto the outstretched hand.

For displaced fractures that meet surgical criteria, the traditional deltopectoral (DP) approach is the most common approach for plate fixation of proximal humeral fractures. However, some authors have argued that this approach involves extensive soft-tissue dissection and muscle retraction to gain adequate exposure to the lateral aspect of the humerus [5]. As an alternative, a less invasive deltoid-split (DS) approach has been described with the goal of minimizing local soft-tissue trauma [6,7].

Only a few nonrandomized trials have compared these two approaches in the literature so far [8,9,10,11]. Operation time, intraoperative blood loss, early complications, and postoperative anatomical reduction were defined as the perioperative parameters. There are no scientific studies comparing all these parameters in comminuted proximal humerus fractures, especially the postoperative anatomical reduction using the DS and DP approaches in Neer III and IV type fractures.

In light of the deficiencies in the existing evidence base, we conducted a randomized controlled trial. Our aim in this study is to compare the two approaches in terms of intraoperative parameters such as operative time and blood loss during the surgery and final reduction achieved.

## Materials and methods

Ethical approval

Dr. D. Y. Patil Medical College Institutional Ethics Committee approved the study with the approval number I.E.S.C/206/2018.

Study design and setting

This was a prospective randomized control cohort study conducted from August 2018 to August 2020 at the Dr. D. Y. Patil Medical College, Hospital and Research Centre, Pimpri, Pune. All procedures were carried out by a single operating surgeon with more than 20 years of experience.

Sample size

A total of 46 patients consented to participate in the study during the study period. After applying the exclusion criteria, only 42 patients were included in the final analysis. All postoperative X-rays in two orthogonal planes were assessed by an independent observer.

Inclusion criteria

The inclusion criteria were as follows: all adult patients aged between 20-60 years who consented to participate; patients with three-part or four-part comminuted proximal humerus fractures; patients fit for surgical intervention; patients treated with open reduction and internal fixation (ORIF) with Proximal Humeral Interlocking System (PHILOS) plating.

Exclusion criteria

The exclusion criteria were as follows: patients aged less than 20 years and more than 60 years; patients with distal neurovascular deficits; patients presenting with compound fractures; patients with pathological fractures; patients undergoing other treatment methods such as nailing, percutaneous K-wire fixation, and conservative methods (nonoperative); terminally ill patients with multiple medical comorbidities.

Randomization method

A computer-generated random table was used for randomizing patients into one of the two groups based on the approach employed, which was communicated to the operating surgeon one day prior to the surgery.

Preoperative patient evaluation

All patients were evaluated with routine blood investigations and a cardio-respiratory assessment was obtained prior to the surgery. In the ward, a preoperative wash was given with Betadine scrub solution. Also, 1.5 g of injection cefuroxime was given intravenously.

Preoperative Radiographic Evaluation

Fractures were evaluated using AP and axillary views of the shoulder. The axillary view was used to determine the relationship of the humeral head with the glenoid, as well as to outline the lesser and greater tuberosity displacement. The axillary view was also used to identify the posterior retraction of the greater tuberosity in three-part fractures [6].

Patients were kept in a supine position with a sandbag beneath the spine and the medial border of the scapula to move the affected side forward while allowing the arm to drop backward, opening up the front of the joint. To relieve venous pressure and allow blood to flow away from the operation site, the head end of the table was raised by 30-45°. The arm was fully draped to allow it to move freely during surgery. The patients were given general anesthesia.

Deltopectoral approach

**Figure 1:**
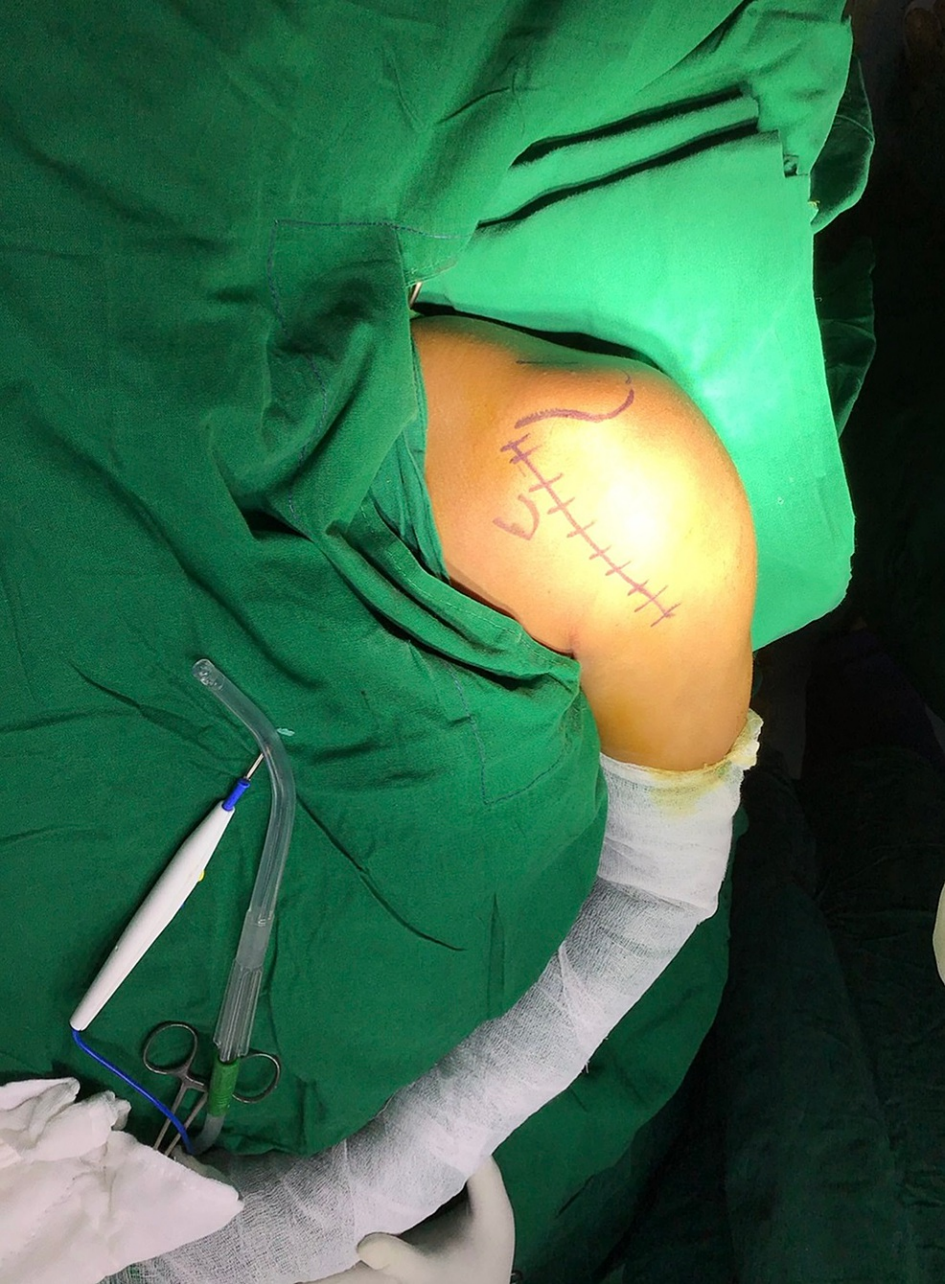
Deltopectoral incision marking with the coracoid process and acromion process marking

**Figure 2:**
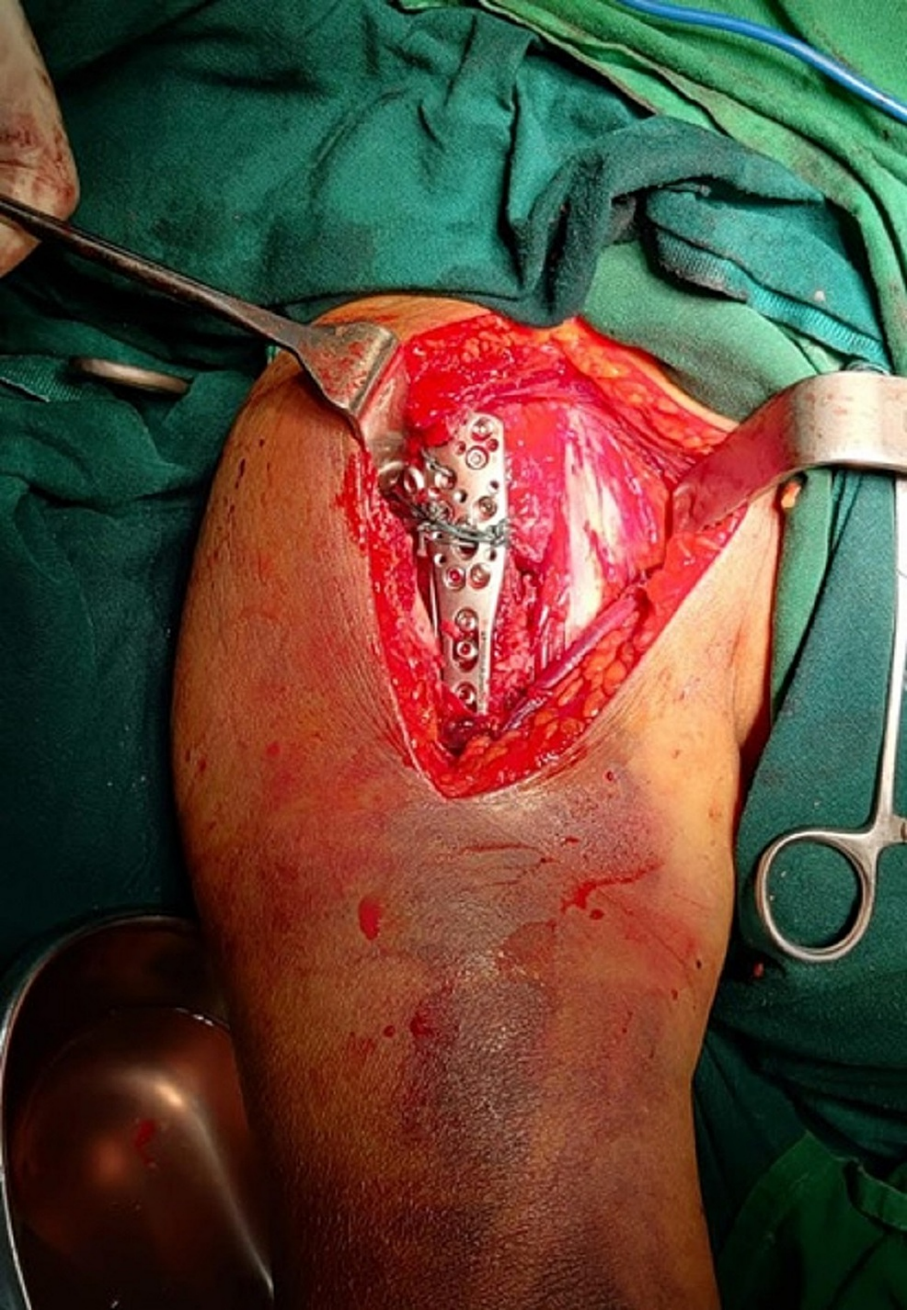
PHILOS plate application through the deltopectoral approach PHILOS: Proximal Humeral Interlocking System

**Figure 3:**
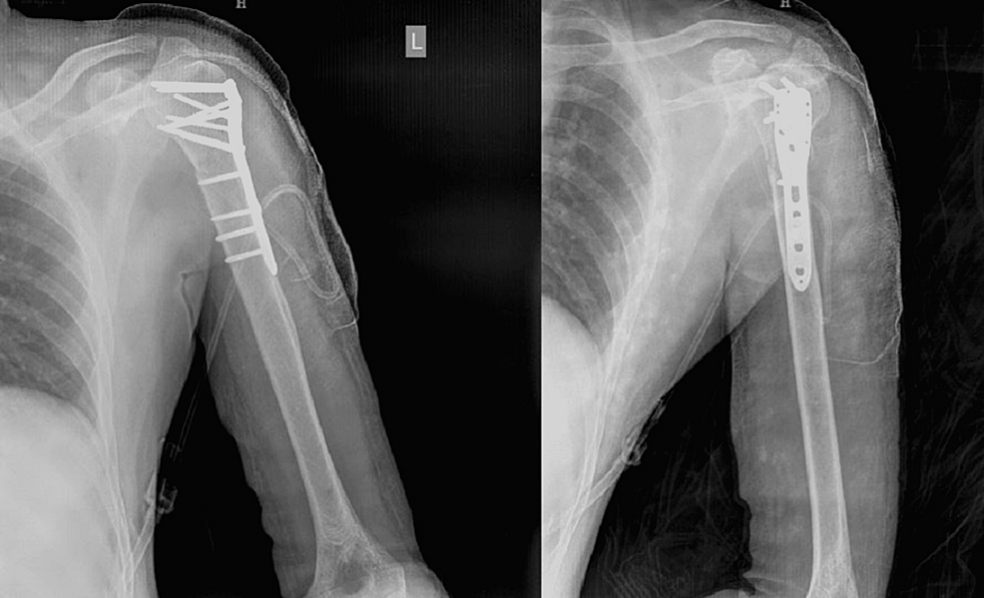
Postoperative X-rays of PHILOS fixation for proximal humerus fracture through the deltopectoral approach PHILOS: Proximal Humeral Interlocking System

The images that illustrate the standard DP approach are given below (Figures 1-3) [12,13,14].

Deltoid-split approach

**Figure 4:**
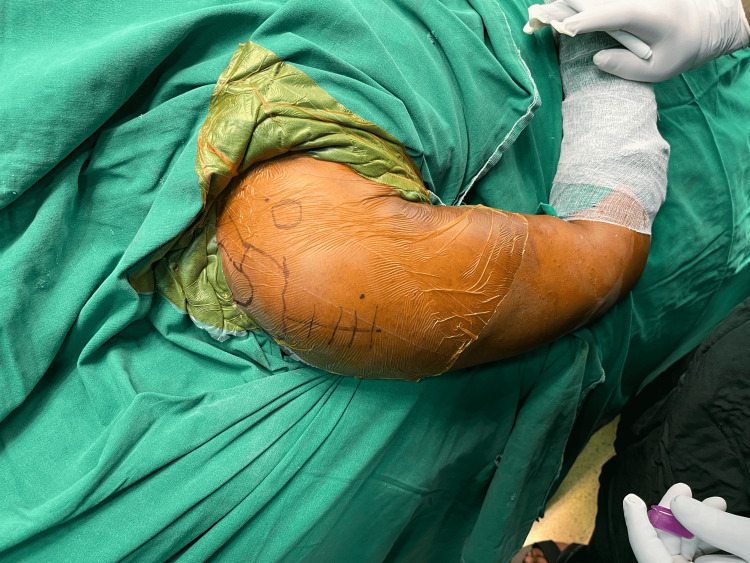
Deltoid-split incision marking lateral to the acromion process

The incision marking in the DS approach is depicted in Figure 4.

Distally, in line with the arm, a 5-cm incision is made from the acromion point. This is usually done around the clavicle's posterior edge; however, it can be moved about depending on the pathology [12,14].

Superficial Dissection

To safeguard the axillary nerve, the deltoid muscle is incised in line with its muscle fibers no more than 5 cm distal to the lateral margin of the acromion. To prevent the split from propagating, a stay suture is put at the inferior apex [12,13,14].

Deep Dissection

The subacromial bursa is located directly beneath the deltoid muscle and can be resected to reveal the rotator-cuff insertion and proximal humerus beneath [12,13,14].

Extension of the Deltoid-Split Approach

The distal extension is only possible if a second, discrete DS is performed distal to the axillary nerve. The approach is extended proximally parallel to the scapula's spine to expose the whole supraspinatus. The scapula's spine must be divided parallel to the overlying trapezius muscle, and the acromion must be divided in line with the incision, both of which must be mended.

**Figure 5:**
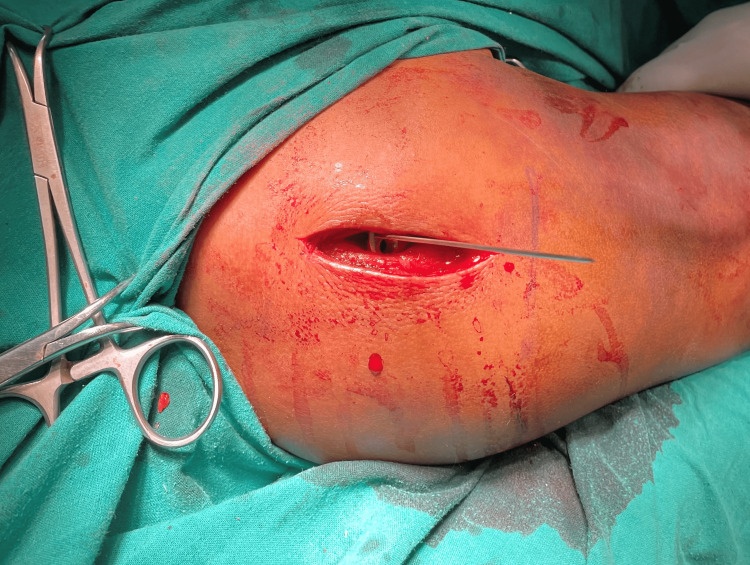
Deltoid-split approach deep dissection showing the axillary nerve in the bottom

Figure 5 depicts the deep dissection in the DS approach showing the axillary nerve in the bottom.

Fracture fixation

**Figure 6:**
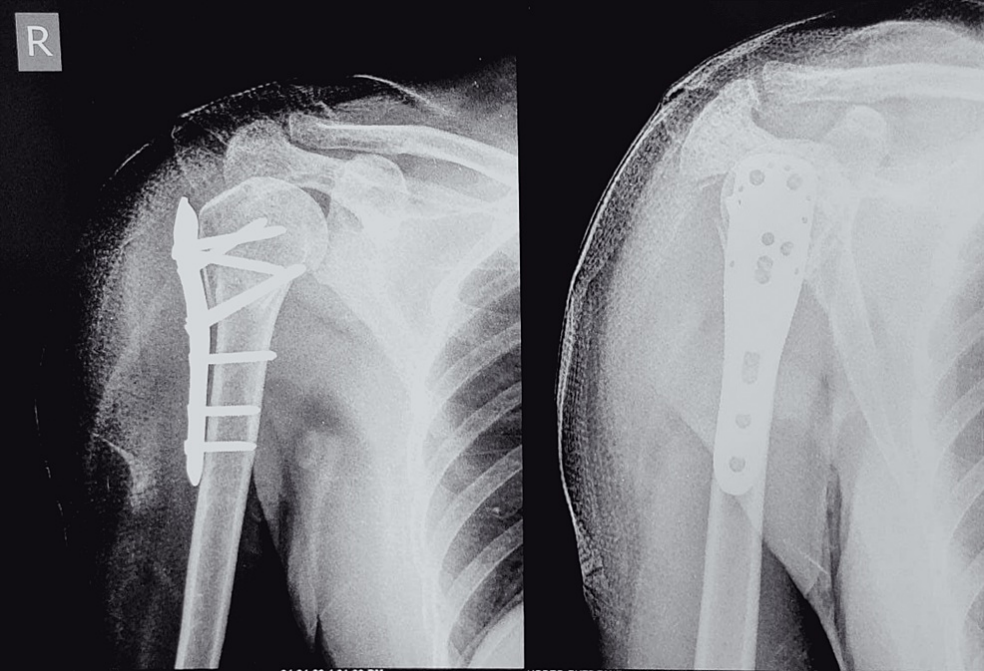
Anteroposterior and lateral images of postoperative PHILOS plate fixation in proximal humerus fixation through the deltoid-split approach PHILOS: Proximal Humeral Interlocking System

The remainder of the surgical techniques when applied did not vary between the two groups. On reaching the fracture site, fracture parts were reduced to the best feasible anatomical position without removing the periosteum and provisionally fixed to the head fragment with a K-wire. After analyzing the reduction with an image intensifier, the PHILOS plate was definitively fixed, with the plate positioned at least 5 mm distal to the upper end of the larger tuberosity, protecting the long head of the biceps tendon. After that, locking cancellous screws were utilized to fix the proximal pieces and cortical screws were used to fix the shaft, all the while maintaining the reduction of the fracture. Tuberosities were repaired to the plate by utilizing the tension band technique and absorbable sutures whenever they were found fractured [12,13]. Figure 6 shows anteroposterior and lateral images of postoperative PHILOS plate fixation with the DS approach.

Analysis

**Figure 7:**
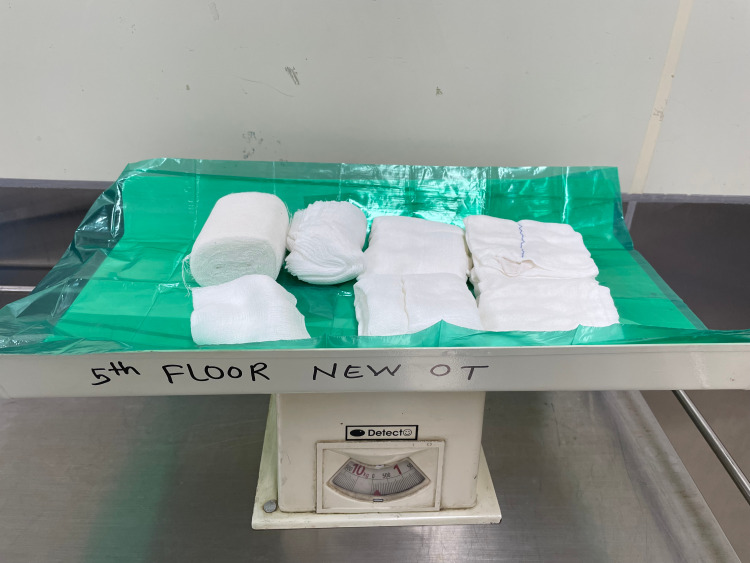
Preoperative weighing of the mops used in surgery

**Figure 8:**
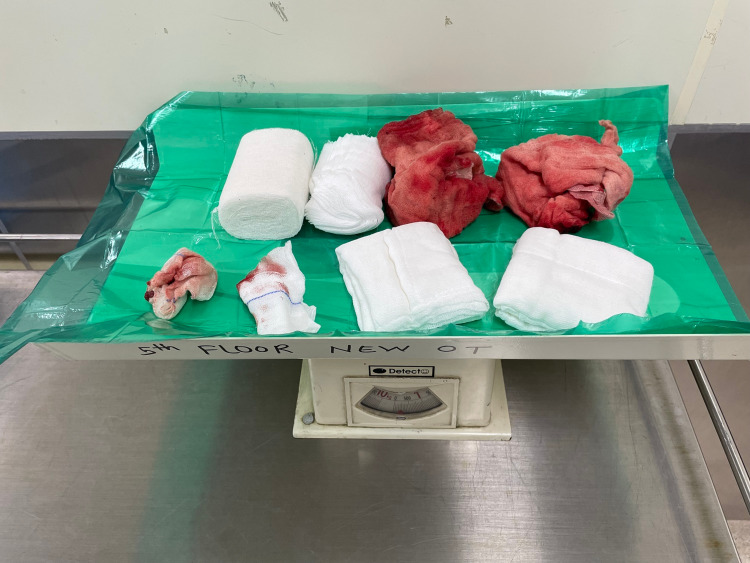
Postoperative weighing of the mops used in surgery

Perioperative parameters such as time taken for surgery, intraoperative blood loss, and immediate postoperative complications were noted. Time taken for surgery was defined as the time from incision to skin closure noted intraoperatively over the timing counter. Intraoperative blood loss was measured using the gravimetric method where principally 1 gram of weight is equal to 1 ml of blood loss. The weight of lost blood was calculated from the suction fluid in the canister, excluding the amount of fluid given for wash as well as the difference between the weight of mop pads preoperatively and postoperatively. Precautions were taken not to spill the blood on the floor and draping was done using nonabsorbable sterile plastic drapes [15]. Figure 7 displays the preoperative weighing of the mops used in surgery. Figure 8 shows the postoperative weighing of mops used in surgery. Their difference was used to assess the blood loss.

Blood loss = (suction volume in the canister - the amount of wash fluid given) in ml + (difference in the weight of mops pads in grams) ml [15].

Radiographic assessment of anatomical reduction

**Figure 9:**
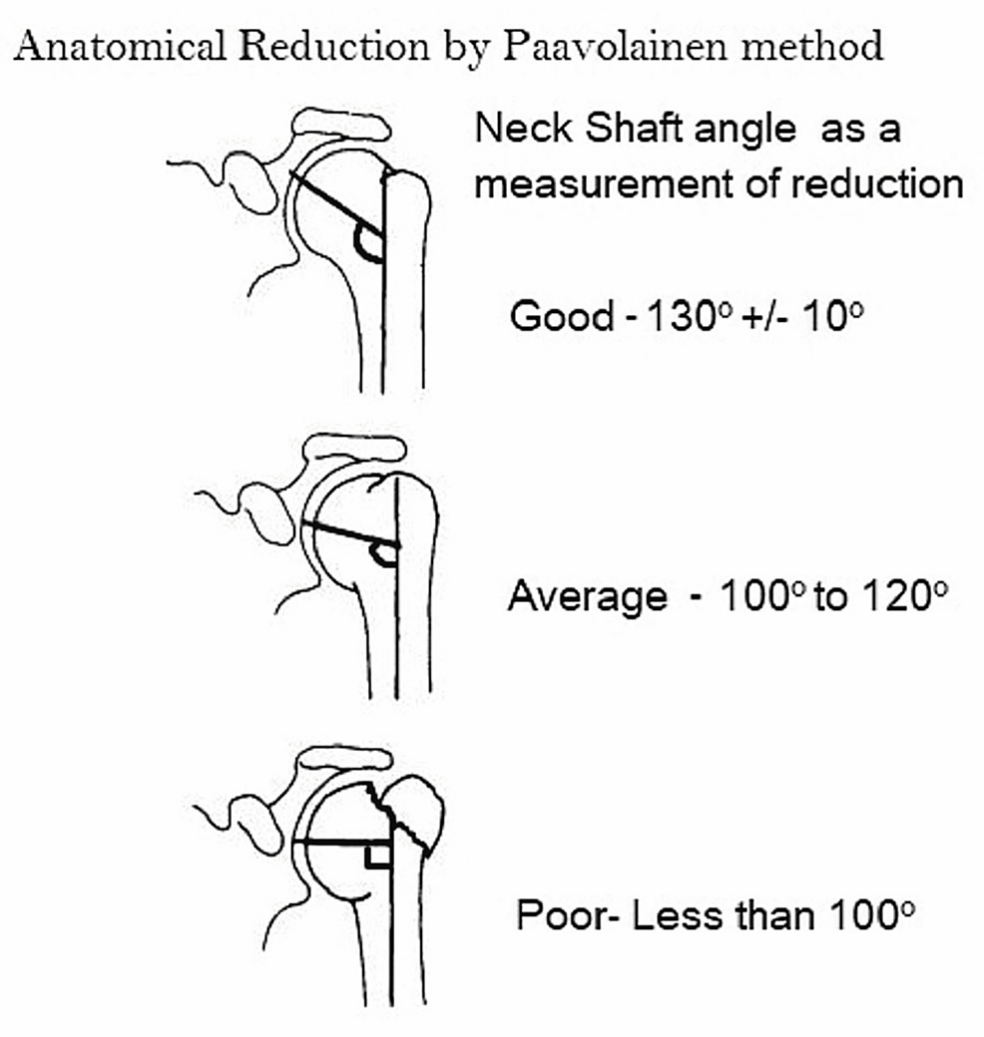
Anatomical reduction as measured by Paavolainen method in anteroposterior view of the shoulder in postoperative X-ray* *[16]

The AP X-ray should show the correct relationship between the humeral head and the tuberosities. The fracture position was rated as good if exact fracture alignment and correct inclination of the humeral neck (130^ ^± 10^o^) were combined with good positioning of the implants; as average if the inclination of the humeral neck was 100-120^o^; and as poor if the inclination was less than 100^o^. Figure 9 illustrates anatomical reduction as measured by the Paavolainen method in AP view of the shoulder in postoperative X-ray.

Postoperative rehabilitation

In both groups, postoperative rehabilitation was performed in a similar fashion. For the first two days after surgery, the operated shoulder was kept in a universal shoulder immobilizer. Then came the start of early passive and limited active movements for the initial three weeks. For the first six weeks after surgery, only limited aided abduction up to 90 degrees was allowed in patients with higher tuberosity fractures (e.g., three- and four-part fractures) [12,17,18].

Statistical analysis

The Chi-square test was used for categorical data for the two groups. The t-test was used for continuous variables and the independent t-test was used for the comparison of two groups. For age and blood loss, the distribution was skewed; therefore, we used an independent sample median test to compare the two methods. A p-value of less than 0.05 was considered statistically significant. The SPSS Statistics software (IBM Corp., Armonk, NY) was used for the statistical analysis.

## Results

All patients' demographic data (gender, age, and fracture type) were gathered. All fractures were classified using preoperative radiographs according to the Neer approach. Perioperative information on the basis of documentation sheets and postoperative X-rays, all data relevant to the hospital course (operative time, intraoperative blood loss, and immediate postoperative complications) as well as final surgical restoration of humerus anatomy were recorded.

The present study included 42 cases of proximal humerus fractures in adults treated by ORIF with PHILOS plating. Of the 42 cases, 22 cases were fixed using the DS approach, and the DP approach was used for the remaining 20. The majority of the cases of proximal humerus fractures belonged to the age group of 41-50 years, with the mean age being 47.52 ± 10.77 years. There was no significant statistical difference between the groups in terms of age (p=0.757) (Table 1).

In the DS group, 14 patients had three-part fractures and eight patients had four-part fractures. Similarly, in the DP group, 12 patients had three-part fractures and eight patients had four-part fractures. There was no significant difference between the groups with regard to fracture patterns (p=0.808) (Table 1).

In the DS group, seven patients had right-sided fractures and 15 patients had left-sided fractures, whereas in the DP approach, 12 patients had right-sided fractures and eight patients had left-sided fractures, with no statistically significant difference (p=0.06). The DS group had 12 males and 10 females, while the DP group had nine males and 11 females, with no statistically significant difference between the groups (p>0.54) (Table 1).

Of the 42 cases treated by ORIF with PHILOS plating, 22 cases in the DS-approach group had an average blood loss of 158 ml whereas 20 cases in the DP approach had an average blood loss of about 227.75 ml. The combined average blood loss in both groups was 191.31 ± 36.86 ml. The difference between the groups was not found to be statistically significant (p>0.575) (Table 1).

The 22 cases in the DS group had an average operation time of 98.86 minutes whereas the 20 cases in the DP group had an average surgical time of about 110 minutes. The average surgical time for all cases combined was 104.17 ± 37.76 minutes; the difference was not found to be statistically significant (p>0.26) (Table 1).

**Table 1:** Statistical analysis of differences between DS and DP groups based on different variables DS: deltoid-split; DP: deltopectoral; NS: not significant; SD: standard deviation

Variable	Subtypes	DS approach [n=22 (52.38%)], n (%)	DP approach [n=20 (47.62%)], n (%)	Total [n=42 (100%)], n (%)	P-value
Fracture pattern	Neer type 3	14 (63.64)	12 (60)	26 (0.620)	0.808
	Neer type 4	8 (36.36)	8 (40)	16 (0.38)	
Gender	Male	12 (54.54)	9 (45)	21 (0.5)	0.54 (NS)
	Female	10 (45.45)	11 (55)	21 (0.5)	
Side	Right	7 (31.82)	12 (60)	19 (0.45)	0.06 (NS)
	Left	15 (68.18)	8 (40)	23 (0.55)	
		Mean	Mean	Mean ± SD	
Age, years		49.14	45.75	47.52 ± 10.77	0.757 (NS)
Total operation time, minutes		98.86	110	104.17 ± 37.76	0.26 (NS)
Intraoperative blood loss, ml		158.18	227.75	191.31 ± 136.86	0.575 (NS)

Of note, significantly more patients (p=0.032) who underwent DP-approach surgery showed good anatomical restoration compared to patients who underwent DS surgery (55% vs. 27%) in comparison with average and poor anatomical restoration (40% vs. 36%) (Table 2).

**Table 2:** Comparison of the bony anatomical restoration by the Paavolainen method between DS and DP approaches DS: deltoid-split; DP: deltopectoral

Anatomical restoration of fractures by Paavolainen method	DS approach, n (%)	DP approach, n (%)	Total
Good	6 (27.27)	11 (55)	17
Average	8 (36.36)	8 (40)	16
Poor	8 (36.36)	1 (5)	9
Total	22 (52.38)	20 (47.62)	42
P=0.032			

There was a case of axillary nerve injury in the DS group, which probably occurred due to excess traction in an attempt to reduce a four-part fracture. Also, a case of subacromial impingement was noted in the DS group postoperatively. A case of screw penetration into the glenohumeral joint was noted in the DP group. Additionally, a case of varus collapse was noted in the DP group in the immediate postoperative X-ray (Table 3).

**Table 3:** Incidence of complications in the study population DS: deltoid-split; DP: deltopectoral

Type of complication	DS approach	DP approach
Axillary nerve injury	1 case	Nil
Subacromial impingement	1 case	Nil
Screw penetration into glenohumeral joint	Nil	1 case
Varus collapse	Nil	1 case
Total	2 cases	2 cases

## Discussion

This prospective randomized study proposes to compare DS and DP approaches in the ORIF of proximal humeral fractures with a PHILOS plate in terms of operative time, blood loss, and early and immediate complications of surgical fixation.

The patients in both surgical approach groups were matched and comparable with regard to age and gender. According to the Neer classification, 22 patients had three and four-part fractures in the DS group and 20 in the DP group. Hence, we matched the patient-specific and fracture-specific parameters as much as possible. However, there was no differential loss to follow-up between the study groups as all the cases that fulfilled the inclusion and surgical criteria were included. A computer-generated random table was employed for randomizing patients into one of the two groups based on the approach employed. We excluded those patients treated with other modalities such as conservative management, treatment with K-wires, hemiarthroplasty, and total shoulder arthroplasty. Hence, we could not perform an analysis on the intention to treat [18].

In their study, Buecking et al. found that the operating time in the DS group was significantly lower than that in the DP group (SMD: -0.89, 95% CI: -1.27 to -0.52; p=0.001) using the fixed-effect model [8]. Though the DS approach is quicker in most of the cases, extremes are encountered in certain comminuted four-part cases treated by the DS approach where manually attaining reduction is difficult. Kumar et al. found similar results in a systemic review with less sampling error [11]. In our study, the mean total intraoperative time taken was lower in the DS group compared to the DP group (p>0.101), but the difference was not statistically significant; however, there were extremes in both the groups due to the complexity of fractures. Though there were some cases where the operative time was much longer than the rest of the cases in both groups, the mean operative time difference among both groups did not reach statistical significance. Shorter operative time among a few cases of the DS group corresponded to fracture patterns involving greater tuberosity avulsion along with surgical neck of the humerus with minimal displacement.

Zyto et al. found that there was a significant difference in terms of blood loss favoring the DS group [1]. Volumetric analysis of blood loss was the reproducible method employed to assess the rough amount of blood loss though all the blood exsanguinated could not be accounted for due to its absorption into surrounding drapes and other losses [12]. Though muscle bleeding was present in the DS approach, in our study, blood loss was more frequently observed in comminuted fractures and in those operated with the DP approach (231 ml). The chances of bleeding from the accidental rupture of the cephalic vein were present in a few cases, which increased the mean blood loss. In contrast, the DS group showed less blood loss (158 ml) owing to no major manipulations around the fracture sites to obtain reduction.

Minkus et al. noted that anatomical reduction with the restoration of the medial calcar is important for vascularization and to prevent a secondary dislocation. Restoration of anatomy has taken into consideration the humeral neck-shaft angle reduction to near normal, to about 130° [18]. Xie et al. in their meta-analysis found that there was no significant difference between the two groups (RR: 0.68, 95% CI: 0.12-4.03), with no heterogeneity (I^2^=0%, p=0.94) [3]. Fracture reduction with minimal distortion of the fragments and avoidance of screw penetration into glenohumeral joint space result in a good outcome. All the immediate postoperative X-rays were assessed by a single third-party observer who was a senior consultant. The surgery in cases of greater tuberosity avulsion was straightforward with the fixation of fractured process on the bone with a plate over it, and no major manipulations were involved. We also found that the use of a curved Hohmann retractor can help the surgeon in viewing and achieving a reduction in the DS group while Sauerbruch retractors are sufficient in the DP group as the approach is quite extensile in both the directions.

Solberg et al., in 2009, reported that 70% of their patients with preoperative varus alignment still had at least 5° of varus after undergoing fixation [19]. In our study, we found complications such as axillary nerve injury and subacromial impingement in the DS group, whereas screw penetration in the glenohumeral joint and varus collapse were observed in the DP group. Although not statistically significant because of fewer numbers, we found a different distribution of complications between the DS and the DP groups.

Limitations of the study

The blood-loss calculation measure was based on the gravimetric method, which is the best method for orthopedic surgery blood-loss calculations. However, some blood loss due to seepage into the drapes could not be calculated, but the use of plastic drapes along with iodine-coated skin drapes minimized these errors. The study was conducted in a limited timeframe at a single center. Hence, we recommend multi-centric studies with larger sample sizes to achieve a more significant impact value.

## Conclusions

There were no statistically significant differences between the DP and DS groups in terms of variables such as blood loss, time taken for surgery, and immediate postoperative complications. However, with regard to the postoperative reduction achieved as per the Paavolainen method of assessment, the DP group demonstrated better, statistically significant results (p=0.032) when compared to the DS group.

## References

[1] Zyto K, Ahrengart L, Sperber A, Törnkvist H (1997). Treatment of displaced proximal humeral fractures in elderly patients. J Bone Joint Surg Br.

[2] Kishore JV, Tonape P (2020). Clinical and functional outcome of proximal humerus fractures treated with locking compression plate (LCP) in adults-a prospective study. Indian J Orthop.

[3] Xie L, Zhang Y, Chen C, Zheng W, Chen H, Cai L (2019). Deltoid-split approach versus deltopectoral approach for proximal humerus fractures: a systematic review and meta-analysis. Orthop Traumatol Surg Res.

[4] Aggarwal S, Bali K, Dhillon MS, Kumar V, Mootha AK (2010). Displaced proximal humeral fractures: an Indian experience with locking plates. J Orthop Surg Res.

[5] Johnson NA, Pandey R (2019). Proximal humerus fracture-dislocation managed by mini-open reduction and percutaneous screw fixation. Shoulder Elbow.

[6] Röderer G, Abouelsoud M, Gebhard F, Böckers TM, Kinzl L (2007). Minimally invasive application of the non-contact-bridging (NCB) plate to the proximal humerus: an anatomical study. J Orthop Trauma.

[7] Ruchholtz S, Hauk C, Lewan U, Franz D, Kühne C, Zettl R (2011). Minimally invasive polyaxial locking plate fixation of proximal humeral fractures: a prospective study. J Trauma.

[8] Buecking B, Mohr J, Bockmann B, Zettl R, Ruchholtz S (2014). Deltoid-split or deltopectoral approaches for the treatment of displaced proximal humeral fractures?. Clin Orthop Relat Res.

[9] Bahrs C, Kühle L, Blumenstock G, Stöckle U, Rolauffs B, Freude T (2015). Which parameters affect medium- to long-term results after angular stable plate fixation for proximal humeral fractures?. J Shoulder Elbow Surg.

[10] Rouleau DM, Balg F, Benoit B, Leduc S, Malo M, Vézina F, Laflamme GY (2020). Deltopectoral vs. deltoid split approach for proximal HUmerus fracture fixation with locking plate: a prospective RAndomized study (HURA). J Shoulder Elbow Surg.

[11] Kumar S, Mishra A, Singh H, Clark D, Espag M, Tambe A (2021). Surgical fixation of isolated greater tuberosity fractures of the humerus- systematic review and meta-analysis. J Clin Orthop Trauma.

[12] De Boer P, Buckley R, Hoppenfeld S (2021). Surgical Exposures in Orthopaedics: The Anatomic Approach. http://lww.com/Surgical-Exposures-in-Orthopaedics--The-Anatomic-Approach/p/9781975168797.

[13] (2022). ORIF - Plate fixation for 4-Part, slight displacement, valgus malalignment. https://surgeryreference.aofoundation.org/orthopedic-trauma/adult-trauma/proximal-humerus/4-part-slight-displacement-valgus-malalignment/orif-plate-fixation#plate-fixation.

[14] McKean JM, Seligson D (2015). Upper limb anatomy and surgical approaches. Passport for the Orthopedic Boards and FRCS Examination.

[15] Lee MH, Ingvertsen BT, Kirpensteijn J, Jensen AL, Kristensen AT (2006). Quantification of surgical blood loss. Vet Surg.

[16] Paavolainen P, Björkenheim JM, Slätis P, Paukku P (1983). Operative treatment of severe proximal humeral fractures. Acta Orthop Scand.

[17] Fidanza A, Rossi C, Iarussi S, Necozione S, Indelli PF, Calvisi V (2021). Proximal humeral fractures treated with a low-profile plate with enhanced fixation properties (Epub ahead of print). J Orthop Sci.

[18] Minkus M, Scheibel M (2019). Open reduction, retention, and fixation of proximal humeral fractures using a locking plate osteosynthesis. Obere Extremität.

[19] Solberg BD, Moon CN, Franco DP, Paiement GD (2009). Locked plating of 3- and 4-part proximal humerus fractures in older patients: the effect of initial fracture pattern on outcome. J Orthop Trauma.

